# Prevalence of Tobacco Use Among Power Loom Workers - A Cross-Sectional Study

**DOI:** 10.4103/0970-0218.62551

**Published:** 2010-01

**Authors:** Zaki Anwar Ansari, S Nafees Bano, M Zulkifle

**Affiliations:** Department of Preventive and Social Medicine, A.F.U.M.C., Indore, India; 1 Department of Obstetrics and Gynecology, A.F.U.M.C., Indore, India; 2 Preventive and Social Medicine, NIUM, Bangalore, India

**Keywords:** Tobacco chewing, tobacco smoking, power loom workers

## Abstract

**Background:**

Tobacco use is a major public health problem globally. According to the World Health Organization (WHO), tobacco is the second most important cause of death in the world. It is currently estimated to be responsible for about 5 million deaths each year worldwide. In India, it is responsible for over 8 lakh deaths every year.

**Objective:**

To estimate the prevalence of tobacco use among power loom workers in Mau Aima Town, District Allahabad, UP.

**Materials and Methods:**

Five hundred power loom workers were randomly chosen. Out of them 448 workers were interviewed through a questionnaire survey during May-June 2007. Data on demographics, education, and type of work were collected along with details regarding tobacco use and smoking status, duration of the habit, and daily consumption. Prevalence of tobacco chewing and/or bidi and cigarette smoking, and their sociodemographic correlates, were examined.

**Results:**

The overall prevalence of tobacco use was 85.9%; the prevalence of smoking and tobacco chewing were 62.28% and 66.07%, respectively. Statistical analysis showed that smoking is more common in the elderly, while chewing *gutka* (a type of chewing tobacco) is popular among the younger age-groups.

**Conclusion:**

The prevalence of tobacco use among power loom workers is very high compared to that in general population. Immediate intervention programs are warranted to reduce the future burden of tobacco-related morbidity among these workers who are already exposed to the highly polluted environment in power loom factories.

## Introduction

Tobacco use is one of the important preventable causes of death(1) and a leading public health problem all over the world.(2) According to the WHO, tobacco is the second major cause of death worldwide and is currently responsible for about 5 million deaths each year.(3) This figure is expected to rise to about 8.4 million by the year 2020, with 70% of those deaths occurring in developing countries.(4) Eighty-two percent of the world's 1.1 billion smokers now reside in low- and middle-income countries where, in contrast to the declining consumption in high-income countries, tobacco consumption is on the rise.(5) In India, tobacco use is estimated to cause 800,000 deaths annually.(6) The WHO predicts that tobacco deaths in India may exceed 1.5 million annually by 2020.(7)

Tobacco use is harmful and addictive. All forms of tobacco cause fatal and disabling health problems throughout life. Scientific evidence has linked tobacco use with the development of more than 25 diseases. Smoking tobacco is the major cause of lung cancer, chronic obstructive pulmonary disease (COPD), peripheral vascular disease, and various throat and mouth cancers. Tobacco smoking is a known cause of stroke, coronary heart disease, bladder cancer, aortic aneurysm, perinatal mortality, cervical cancer, and leukemia. Oral smokeless tobacco is associated with precancerous lesions and cancers of the oral cavity. In addition to the increased risk for developing these specific diseases, tobacco users have a significantly higher risk for general health problems than nonsmokers.(8)

According to Unani experts, tobacco consumption adversely affects the heart and brain; it can especially affect people having excitable temperaments, causing diseases such as headache, vertigo, loss of memory, insomnia, loss of vision, cough, pulmonary tuberculosis, palpitation, impotence, constipation, etc.(9–13)

Tobacco is used in different forms. Smoking is through cigarettes, *bidis*, *hukka*, and *chilam* (*ganja*). Smokeless tobacco products include tobacco that is used in *pan*, *gutkha*, *zarda*, *khaini*, and *dohra*.

Tobacco use is influenced by a variety of factors, including individual attitudes and beliefs, social norms and acceptability, availability, and advertising campaigns.(8) There are many misperceptions with regard to tobacco use, for example that it aids concentration, suppresses appetite, reduces anxiety and tension, causes skeletal muscle relaxation, and induces feelings of pleasure. Partly as a result of these perceived benefits tobacco consumption is highest in the labor classes and among those from a low socioeconomic status. Several studies have shown that tobacco use is higher among the less educated or illiterate, and the poor and marginalized groups.(8,14,15)

The present study was done to determine prevalence of tobacco use in a vulnerable population, i.e., power loom workers.

## Materials and Methods

The present study was designed to find out the prevalence of tobacco use among power loom workers in Mau Aima Town, District Allahabad. This study was conducted over the period May 2007-June 2007. Mau Aima is a town and a Nagar Panchayat in Allahabad district in the Indian State of Uttar Pradesh. As per the 2001 Indian census,(16) Mau Aima has a population of 17,962. The main source of income in the study area is the power loom industry.

The sample size was calculated keeping in mind, level of the confidence interval 95%, precision 5% and the calculated existing population 19500. As per our calculations the minimum sample size required was 377 workers. Assuming a response rate of 75%, we randomly selected 500 power loom workers for interview via the pretested structured questionnaire. Informed consent was obtained from the owners of the industries.

Data was collected on age, sex, sociodemographic profile of the workers, consumption of tobacco, age of initiation of habit, reason for initiation, money spent on the purchase of tobacco, frequency of consumption, and form of tobacco used, etc. Tobacco use was classified as current use (initiation of tobacco use within 6 months preceding the survey) and chronic use (the use of tobacco for more than 6 months).

The data were analyzed with the GraphPad InStat Demo version 3.00 for Windows (GraphPad Software, San Diego, CA, USA). Standard methods were used to obtain summary statistics such as median, mean, and standard deviation. The Chi-square test or Fisher's exac*t test* was used for statistical analysis.

## Results

A total of 448 workers responded and were included in the study for analysis. The mean age of the workers was 39.77 ± 11.462 (SD) years. All the power loom workers were male. Fifty-one (11.38%) workers were unmarried; the rest were married or widowed.

**Figure 1:**
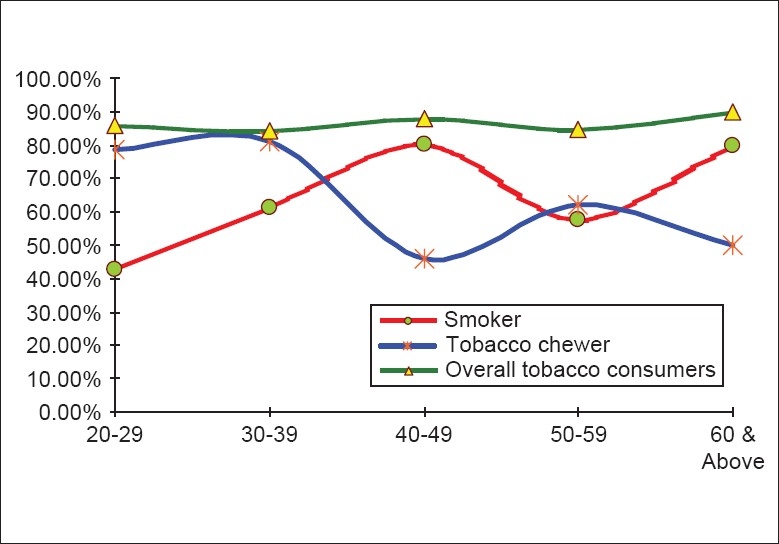
Relation between age and tobacco consumption

The overall prevalence of tobacco use was 85.9% (385/448), while the prevalence of smoking and tobacco chewing were 62.28% (279/448) and 66.07% (296/448), respectively. Amongst all tobacco users, 23.12% (89/385) workers were only smokers, 27.53% (106/385) used only chewing tobacco, and 49.35% (190/385) workers used both [Table 1]. Association between age and overall tobacco consumption was not statistically significant (Chi= 1.109, *P* = 0.8929) as shown in Table 2 and Figure 1. Smoking prevalence was highest in those aged 40-49 years and lowest in those aged 20-29 years, whereas tobacco chewing was highest in those aged 30-39 years and lowest in 40-49 years [Table 2]. This difference was statistically significant (Chi = 25.526, *P* < 0.0001).

**Table 1:** Demographic data

Characteristics	Data value (%)
Age	39.77 ± 11.462 years (SD)
Sex	
Male	100
Female	0
Religion	
Hindu	80 (17.86)
Muslim	368 (82.14)
Marital status	
Married	397 (88.62)
Unmarried	51 (11.38)
Mean monthly income	Rs. 2190.63 ± 248.49 (SD)
Education	
Illiterate	55 (12.28)
Primary	180 (40.18)
Middle	126 (28.13)
Secondary	71 (15.85)
Senior secondary	13 (2.90)
Graduation	2 (0.45)
Overall prevalence of tobacco use	385 (85.9)
Smoking	279 (62.28)
Tobacco chewing	296 (66.07)
Smoking + tobacco chewing	190 (42.41)
Overall tobacco consumers	385/448
Current consumer	17 (4.42)
Chronic consumer	368 (95.58)
Never consumer	63 (14.1)
Age of initiation of tobacco consumption	13.3 years (S.D. ± 3.23)
Influencing factor	
Family member	166 (43)
Friends	146 (38)
Advertisements/other sources	73 (19)
Reason for initiating tobacco consumption	
Pleasure	183 (47.6)
Curiosity	113 (29.3)
Peer pressure	89 (23.1)
Duration of tobacco consumption	Median of 24 years (17 at 25^th^ percentile and 29 at 75^th^ percentile)
Tobacco consumption/day	
Tobacco users	5 (3 at 25^th^ percentile and 8 at 75^th^ percentile)
Cigarette users	2 (1 at 25^th^ percentile and 2 at 75^th^ percentile)
*Bidi* users	10 (10 at 25^th^ percentile and 15 at 75^th^ percentile)
Daily expenditure on tobacco	Rs. 8.63 ± 4.58 (SD)
Knowledge about hazards of tobacco use	358 (80)
Desire to quit tobacco	28 (7.3)
Number of subjects reporting that tobacco consumption helps in morning bowel movement	281 (73)
Number of subjects reporting use of tobacco as the first thing in the morning	334 (86.75)

**Table 2:** Age-wise distribution of tobacco consumption

Age group	Smokers (%)	Tobacco chewers (%)	Smoker + Tobacco chewers (%)	Overall tobacco consumers (%)	No tobacco consumer (%)	Total
20-29	42 (42.86)	77 (78.57)	35 (35.71)	84 (85.71)	14 (14.29)	98
30-39	74 (61.16)	98 (81)	70 (57.85)	102 (84.3)	19 (15.7)	121
40-49	86 (80.37)	49 (45.79)	41 (38.32)	94 (87.85)	13 (12.15)	107
50-59	53 (57.61)	57 (61.96)	32 (34.78)	78 (84.78)	14 (15.22)	92
≥ 60	24 (80)	15 (50)	12 (40)	27 (90)	3 (10)	30
Total	279 (62.28)	296 (66.07)	190 (42.41)	385 (85.94)	63 (14.06)	448

The median duration of tobacco use was 24 years (17 at the 25^th^ percentile and 29 at the 75^th^ percentile). We found that 17 (4.42%) workers were current users of tobacco products and 14% (63/448) had never used tobacco in any form.

Amongst the smokers, the percentages of *bidi*, *hukka*, cigarette, and *ganja* smoking was 74.2% (207/279), 25.45% (71/279), 11.47% (32/279), and 6.81% (19/279), respectively. Amongst the tobacco chewers, 57.8% (171/296) were *gutka* users, 29.4% (87/296) used tobacco with *pan*, 8.1% (24/296) used *khaini*, and 4.73% (14/296) were *dohra users* [Table 3].

**Table 3:** Number of persons reporting consumption of tobacco in various forms according to age-group

Form of tobacco consumption	Age group (in years)					Total
	
	20-29	30-39	40-49	50-59	≥60	
Smoking	42	74	86	53	24	279
*Bidi*	38	47	56	45	21	207
Cigarette	11	3	4	6	8	32
*Hukka*	2	14	29	17	9	71
*Ganja*	0	4	7	3	5	19
Tobacco chewing	77	98	49	57	15	296
*Pan*	33	36	21	38	12	140
*Gutka*	47	73	26	18	7	171
*Khaini*	4	3	8	6	3	24
*Dohra*	6	4	1	3	0	14

**Figure 2:**
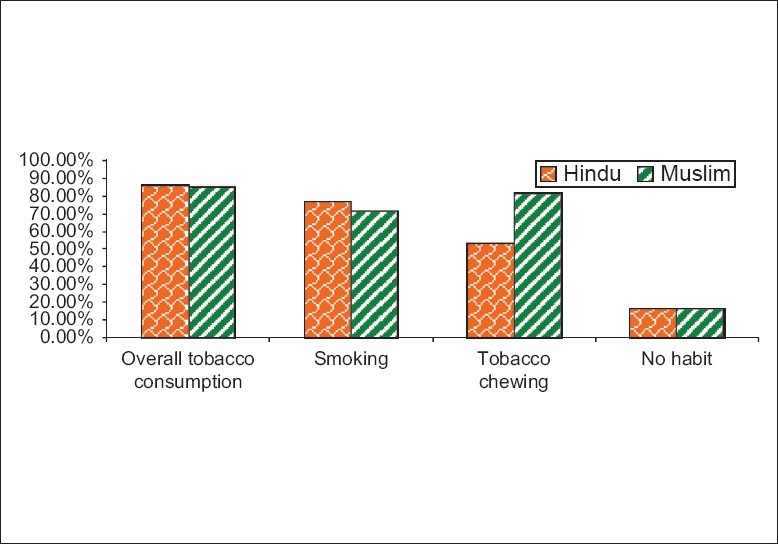
Religion wise distribution of tobacco consumers

Religion-wise distribution of tobacco consumers is shown in Table 4 and Figure 2. In this study, 80 (17.86%) workers were Hindus and 368 (82.14%) were Muslims. Although the overall tobacco consumption was almost the same in Hindus and Muslims, smoking was more common in Hindus, while tobacco chewing was more in Muslims. This difference was statistically significant (Chi = 25.526, *P* < 0.0001).

**Table 4:** Religion-wise distribution of tobacco consumption

Habit	Religion		Total
		
	Hindu (%)	Muslim (%)	
Tobacco consumption	69 (86.25)	316 (85.87)	385
Smoking	53 (76.81)	226 (71.52)	279
Tobacco chewing	37 (53.62)	259 (81.96)	296
Smoking + Tobacco chewing	21 (30.43)	169 (53.48)	190
No habit	11 (15.94)	52 (16.46)	63
Total	80	368	448

**Figure 3:**
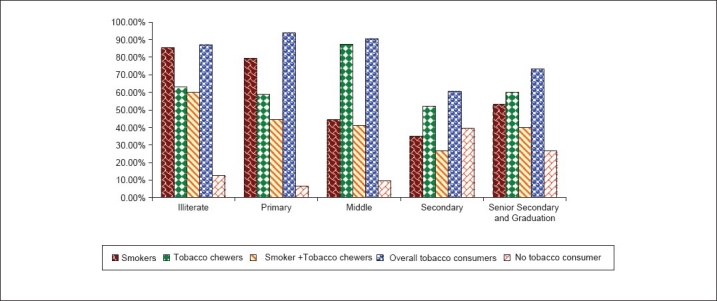
Distribution of tobacco consumers according to education

The relationship between level of education and tobacco consumption is shown in Table 5 and Figure 3. We found that tobacco consumption was more prevalent in those with less education. In general, the education level of power loom workers was very low. Only 0.04% (2/448) were graduates and only 2.9% (13/448) had been educated up to the 12^th^ standard. The majority of the workers (40.4%) had been educated up to the 5^th^ standard. An extremely significant negative association was found between education and tobacco consumption (Chi = 50.301, *P* < 0.0001).

**Table 5:** Tobacco consumption according to educational level

Level of education	Smokers (%)	Tobacco chewers (%)	Smoker + Tobacco chewers (%)	Overall tobacco consumers (%)	No tobacco consumer (%)	Total (%)
Illiterate	47 (85.45)	34 (62.96)	33 (60)	48 (87.27)	7 (12.73)	55 (100)
Primary	143 (79.44)	106 (58.89)	80 (44.44)	169 (93.89)	12 (6.67)	180
Middle	56 (44.44)	110 (87.30)	52 (41.27)	114 (90.48)	12 (9.52)	126
Secondary	25 (35.21)	37 (52.11)	19 (26.76)	43 (60.56)	28 (39.44)	71
Senior secondary and graduation	8 (53.33)	9 (60)	6 (40)	11 (73.33)	4 (26.67)	15
Total	279	296	190	385	63	448

The mean income of workers was Rs. 2190.63 ± 248.49 (SD) per month. Thus, as per the revised Kuppuswamy scale, all workers were of low socioeconomic status [Table 1].

The mean daily expenditure on tobacco was Rs. 8.63 ± 4.58 (SD). Thus, 8.26% (37/448) of the workers spent one-fourth of their income on tobacco products and 27.46% (123/448) workers spent 15-20% of their income for this purpose.

The mean age at initiation of tobacco consumption was 13.3 ± 3.23 (SD) years. Nearly, 48.6% of the workers started using tobacco before the age of 13 years (23.64% at the age of 10 years or below). About 43% of workers reported that they were initiated into the habit by a family member, nearly 38% said that their friends had first introduced them to tobacco use, and 19% said that they had been induced to try out tobacco products by advertisements in various mass media (TV, videos, and movies). Pleasure(47.6%) and curiosity (29.3%) were the two major factors that instigated the consumption of tobacco, while 23.1% of the workers reported that peer pressure was the main reason for starting the habit.

The median use of chewing tobacco was 5 (3 at 25^th^ percentile and 8 at 75^th^ percentile) pouches or quids a day. Cigarette users smoked a median of 2 (1 at 25^th^ percentile and 2 at 75^th^ percentile) cigarettes per day and *bidi* users smoked 10 (10 at 25^th^ percentile and 15 at 75^th^ percentile) per day.

The majority (80%) of the workers knew that tobacco consumption was injurious to health; however, only 28 (7.3%) said that they wished to quit tobacco use.

## Discussion

The aim of this study was to determine the prevalence and type of tobacco use among power loom workers in Mau Aima Town, District Allahabad, and to identify the factors that influenced them to initiate tobacco use.

The overall prevalence of tobacco use in the present study was 85.9%, which is higher than that reported by earlier community-based studies of tobacco use from other parts of the country. In a study of tobacco use in a rural area of Bihar, tobacco use had a prevalence of 78% among men and 52% among women.(17) In a rural community in Khera District in Gujarat, tobacco use was reported by 69% of males and 30% of females.(18) In a prevalence survey of tobacco use in Karnataka and Uttar Pradesh, the overall prevalence of ‘ever use’ of any kind of tobacco was 29.6% in Karnataka and 34.6% in Uttar Pradesh.(2) According to the National Sample Survey Organization, the prevalence rate of tobacco use in the country (rural + urban) is 35.5%.(19)

It is clear from this cross-sectional study that tobacco consumption is highly prevalent among power loom workers. The reasons underlying this may be low educational status, occupation involving hard labor, and poverty. Several studies have documented a positive relationship between tobacco consumption and low socioeconomic status. One major factor for heavy tobacco use in this group may be that they were also doing night shift work.

The educational level of the power loom workers was found to be very low. Consistent with earlier studies, this study found that the lower the education, the higher the prevalence of tobacco consumption. A study of smoking prevalence among men in Chennai (India) in 1997 showed that the highest rate is found among the illiterate population (64%).(20) Hence, education is an important factor to be considered in any tobacco control programme.

The average age at initiation of tobacco use was 13.3 years. About one out of four (23.64%) workers initiated tobacco use at 10 years of age or earlier, which is consistent with earlier studies.(17,21) Though the number of tobacco consumers is almost same in all the age-groups, those who started at an earlier age are more common in the 20-29 and 30-39 age-groups. This data reveals the failure of tobacco control programmes in the vulnerable section of the community.

In this study, we found that 8.26% (37/448) of the workers spent one-fourth of their income and 27.46% (123/448) spent 15-20% of their income on tobacco products. This expenditure on tobacco is very high. Money spent on tobacco means that there is less to be spent on basic human needs such as food, shelter, education, and health care. Tobacco can also worsen poverty among users and their families since tobacco users are at much higher risk of falling ill and dying prematurely of cancers, heart attacks, respiratory diseases, or other such tobacco-related diseases, imposing additional costs for health care and depriving families of much-needed income.(3) Earlier studies have also reported that tobacco consumption increases poverty.(4) In most countries, tobacco use tends to be higher among the poor.(22) The WHO says that in many societies the poorest people tend to smoke the most and bear the greatest health and economic burdens.(23)

## Conclusions

The prevalence of tobacco use among power loom workers is very high compared to that in the general population. Immediate intervention programs are warranted to reduce the future burden of tobacco use-related morbidity among these workers who are already exposed to the high pollution levels in power loom factories.
